# Identification of Novel Protein Biomarkers for Early Detection of Radon-Induced Lung Cancer: A Comparative Study in Kazakhstan

**DOI:** 10.3390/biomedicines14061204

**Published:** 2026-05-27

**Authors:** Baglan Kazhiyakhmetova, Nursulu Altaeva, Meirat Bakhtin, Pavel Tarlykov, Yasutaka Omori, Shinji Tokonami, Chutima Kranrod, Radhia Pradana, Saowarak Musikawan, Anel Lesbek, Danara Ibrayeva, Elena Saifulina, Dana Auganova, Moldir Aumalikova, Madina Kairullova, Aigerim Shokabayeva, Dinara Bizhanova, Yerlan Kashkinbayev

**Affiliations:** 1 Scientific Research Institute of Radiobiology and Radiation Protection NJSC, Astana Medical University, Astana 010000, Kazakhstanaumalikova.m@amu.kz (M.A.); bizhanova.d@amu.kz (D.B.); 2 Department of Medical Genetics and Molecular Biology NJSC, Astana Medical University, Astana 010000, Kazakhstan; 3 National Center for Biotechnology, Astana 010000, Kazakhstan; 4 Institute of Radiation Emergency Medicine, Hirosaki University, 66-1 Hon-Cho, Hirosaki 036-8564, Japan; ys-omori@hirosaki-u.ac.jp (Y.O.); tokonami@hirosaki-u.ac.jp (S.T.);

**Keywords:** radon, lung cancer, protein, biomarkers

## Abstract

**Background:** Radon exposure is the second most important risk factor for lung cancer after tobacco smoking and represents a significant but often underestimated public health problem. Due to the absence of specific clinical manifestations at early stages, the identification of molecular biomarkers reflecting early radon-induced carcinogenic processes is of particular importance. The aim of this study was to identify protein biomarkers associated with radon exposure in lung cancer patients residing in settlements of the Akmola and North Kazakhstan regions of Kazakhstan. **Methods:** Indoor radon exposure was assessed using CR-39 detectors to measure radon concentrations in residential dwellings during summer and autumn periods. The study included 57 lung cancer patients and 73 control subjects residing in areas characterized by varying levels of radon exposure. Plasma samples were collected and analyzed using liquid chromatography–tandem mass spectrometry (LC–MS/MS) to identify differentially expressed proteins associated with lung cancer and radon exposure. Statistical analyses were performed to evaluate differences between groups and associations between radon exposure and molecular biomarkers. **Results:** Seasonal variability in indoor radon concentrations was observed, with several settlements demonstrating levels exceeding international reference values. Proteomic analysis identified multiple proteins differentially expressed between lung cancer patients and controls, as well as between radon-exposed and non-exposed lung cancer patients. Several proteins involved in inflammation, lipid metabolism, oxidative stress, and immune regulation pathways demonstrated significant differences in expression levels, suggesting potential associations with radon-induced carcinogenic mechanisms. LC–MS/MS proteomic profiling identified multiple differentially expressed proteins associated with lung cancer and radon exposure after false discovery rate correction. Proteins involved in inflammation, oxidative stress, immune regulation, and lipid metabolism, including ORM2, AZGP1, PRDX2, IRF7, and APOC3, demonstrated significant expression differences between radon-exposed and low-exposure groups. **Conclusions:** The identified protein biomarkers demonstrated significant associations with both radon exposure and lung cancer status, indicating their potential relevance for early detection and risk assessment of radon-induced lung cancer. The integration of environmental exposure assessment with proteomic profiling may provide new insights into the molecular mechanisms of radon-associated carcinogenesis and support the development of preventive strategies.

## 1. Introduction

Radon (Rn-222) is a naturally occurring, colorless, odorless radioactive noble gas formed as a decay product of uranium-238 present in soils and rocks. Due to its gaseous nature and a half-life of 3.83 days, radon can migrate through porous ground and accumulate in enclosed spaces, particularly in buildings with inadequate ventilation or underground structures. As a result, radon represents the primary source of natural ionizing radiation exposure for the general population worldwide [1].

Epidemiological studies consistently identify radon as the leading cause of lung cancer among non-smokers and the second most significant cause overall after tobacco smoking. According to the estimate by Gaskin et al. [2], radon exposure is responsible for approximately 230,000 lung cancer deaths annually, totaling for 66 countries. Importantly, no safe threshold for radon exposure has been established, as the associated cancer risk increases linearly with concentration, even at levels below current reference values for indoor radon [3,4,5]. Lung cancer remains one of the leading causes of cancer-related mortality worldwide, driven by both lifestyle and environmental risk factors, including air pollution, occupational exposures, and ionizing radiation. In this context, radon represents a particularly important but often underrecognized environmental carcinogen, especially in regions with favorable geological conditions for radon release and limited implementation of radon monitoring and mitigation strategies [6].

Ionizing radiation, including alpha particles emitted by radon progeny, induces molecular alterations involved in carcinogenesis, including DNA damage, oxidative stress, and genomic instability. Previous studies have shown that radon exposure may affect gene and protein expression related to inflammation, apoptosis, and cell proliferation pathways [7]. Proteomic approaches have increasingly been applied to identify molecular biomarkers associated with lung cancer development and radiation response. In addition, genetic susceptibility to lung cancer has been investigated through analysis of single-nucleotide polymorphisms (SNPs) in genes involved in DNA repair, detoxification, and cell cycle regulation [8].

In Kazakhstan, lung cancer constitutes a major public health burden. According to data from the Kazakh Institute of Oncology, 7350 patients are currently registered for lung cancer follow-up care, including 2473 individuals who have survived more than five years after diagnosis. In 2024, 3707 new cases of lung cancer were reported nationwide. During the first seven months of 2025 alone, 2281 new cases were diagnosed, and if current trends persist, the total number of newly identified cases by the end of 2025 is expected to exceed 2024 levels by approximately 5.5% [9].

Kazakhstan is characterized by extensive legacy uranium mining activities and the presence of large uranium tailings repositories, which represent long-term sources of environmental radiation exposure [10]. Recent studies conducted in northern regions of the country have demonstrated pronounced spatial, structural, and seasonal variability in indoor radon progeny concentrations and associated effective radiation doses, particularly in residential buildings located near uranium tailings and open-pit mining sites. Elevated radon levels were most frequently observed in dwellings with underground structures, such as cellars, and during cold seasons characterized by reduced ventilation and prolonged indoor occupancy [11]. Previous environmental radiation surveys conducted in Northern Kazakhstan have identified settlements located near abandoned uranium mining sites, including Saumalkol, as areas with potentially elevated and spatially variable indoor radon exposure [12]. These findings highlight the relevance of radon as an environmental risk factor in this region and underscore the need for studies integrating environmental exposure assessment with biological markers of lung cancer risk [13].

These data underscore the urgent need to investigate environmental contributors to lung cancer risk within the country. Despite the well-established carcinogenicity of radon, data on radon exposure levels and their biological and epidemiological implications remain limited in many regions, including Kazakhstan [1]. Therefore, the present study aims to investigate the role of radon exposure in lung cancer risk by analyzing indoor radon concentration, protein biomarkers, and SNPs of patients with lung cancer and a control group. By integrating environmental, biological, and clinical data, this study seeks to provide new insights into radon-associated lung carcinogenesis and to contribute to evidence-based strategies for cancer prevention.

## 2. Materials and Methods

### 2.1. Study Area

**Figure 1:**
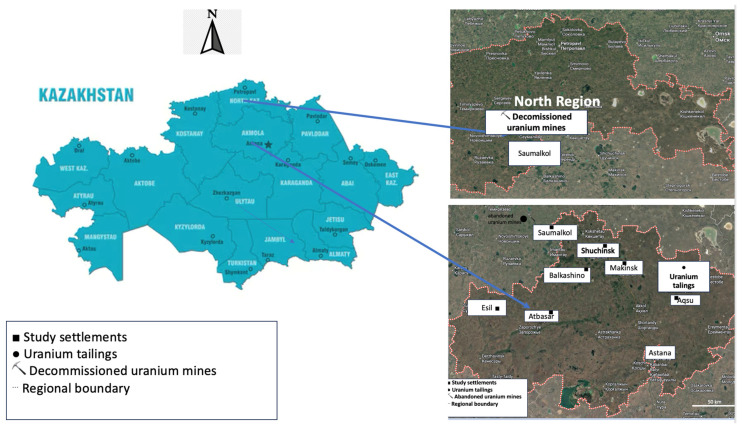
Geographical location of the study area in the Akmola and North Kazakhstan regions of Kazakhstan.

The study was conducted in settlements of the Akmola Region and North Kazakhstan Region, which are characterized by a legacy of uranium mining activities and elevated potential for radon exposure. The settlements were selected based on environmental and infrastructural characteristics associated with increased radon potential and to enable contrasting exposure assessment. Specifically, parts of the study area are located near major uranium-related legacy sites, including tailings dumps, decommissioned uranium mining sites, and regions with ongoing mineral extraction activities. The settlements investigated in the Akmola Region were Aqsu, Makinsk, Burabay, Esil, Atbasar, and Balkashino. The settlement of Aqsu is located approximately 3–4 km from large tailing dumps containing uranium mining waste. In addition, Aqsu is known for its mineral extraction industry, including gold, platinum, palladium, and molybdenum production. The area contains two active open-pit mines, which represent significant sources of radon release into the environment. The population of Aqsu is approximately 4000 residents [11]. The North Kazakhstan Region includes the settlements of Saumalkol and Arykbalyk, both located in proximity to decommissioned uranium mining sites. Saumalkol is situated near a historically significant uranium mining area, while Arykbalyk is located close to an abandoned mine. These settlements are part of a predominantly agricultural region, known for wheat production and livestock farming; however, abandoned industrial sites associated with past uranium extraction pose ongoing environmental and public health challenges [13]. These investigated settlements were selected based on environmental characteristics associated with increased radon potential and according to the findings of an environmental radiation monitoring report conducted by EcoService LLP [14]. The report identified these areas as priority locations for assessing radiation exposure and potential oncological risks due to the presence of uranium mining legacy sites, including tailings storage facilities and abandoned uranium mines. This approach allowed the evaluation of populations residing in areas with contrasting radon exposure levels (high and low) under comparable climatic conditions. At the same time, additional settlements within the same regions were included to represent dwellings with lower indoor radon levels, allowing comparison between high- and low-exposure conditions while minimizing climatic heterogeneity. The geographical distribution of the investigated settlements within the Akmola and North Kazakhstan regions is shown in Figure 1.

### 2.2. Study Population

The study included 57 patients with lung cancer and 73 control subjects permanently residing in the investigated settlements of the Akmola Region and the North Kazakhstan Region. Lung cancer diagnoses were histologically confirmed and obtained from regional oncology hospitals of the Akmola and North Kazakhstan regions. Control participants were recruited from the same geographic areas and had no previous history of malignant disease at the time of enrollment. The duration of residence in the study area ranged from 5 to 40 years, ensuring that the assessed indoor radon levels reflected long-term exposure conditions. Participants were recruited on a voluntary basis and provided written informed consent. Demographic and clinical information (age, sex, smoking status, and residential history) was collected using structured questionnaires and, where applicable, medical records. The study included patients with histologically confirmed primary lung cancer residing in the investigated territories. Inclusion criteria for the lung cancer group were: age > 40 years, confirmed diagnosis of primary lung cancer, and residence in the investigated area for at least 5 years. Exclusion criteria included secondary lung tumors, occupational radiation exposure, pregnancy, and age > 80 years. The control group consisted of apparently healthy individuals residing in the same settlements. Inclusion criteria for the control group included absence of oncological diseases, residence in the investigated territory for more than 5 years, and absence of chronic respiratory diseases. During control group formation, sex, age, and duration of residence were considered to ensure comparability between the study groups. Due to the limited number of individuals meeting the inclusion criteria, the control group was recruited from several settlements within the investigated region characterized by similar environmental and geological conditions. Participant recruitment was performed using a consecutive sampling approach including all available lung cancer patients meeting the study inclusion criteria during the investigation period. Because the study was based on analysis of the entire available population of eligible participants, formal a priori sample size and statistical power calculations were not performed. The larger control group size was used to improve statistical power and increase the precision of between-group comparisons.

### 2.3. Radon Measurement and Exposure Assessment

Assessment of radon exposure was performed using passive-type radon detectors, RADUETs (Radosys Ltd., Budapest, Hungary), to obtain a comprehensive characterization of indoor radon levels in residential buildings located in the investigated settlements. The RADUETs consist of two diffusion chambers equipped with CR-39 solid-state nuclear track detectors incorporated into RADUET passive detectors (Radosys Ltd., Budapest, Hungary) with different air exchange rates, allowing discrimination between radon and thoron concentrations [15]. The analysis of the detectors was carried out at the Institute of Radiation Emergency Medicine of Hirosaki University, Japan. After exposure, CR-39 detectors were chemically etched in 6.25 M NaOH solution at 90 °C for 3 h. Track densities were analyzed using an automated optical counting system. Radon concentrations were calculated using calibration coefficients established in the radon chamber of Hirosaki University, Japan, traceable to secondary radon standards. The RADUETs were calibrated using a radon chamber in Hirosaki University based on comparison with a reference detector traceable to a secondary standard of radon activity concentration in Germany. Detailed descriptions of the calibration procedures, processing of CR-39, and calculation methods have been reported previously [16].

Passive detectors were deployed for long-term monitoring to obtain integrated radon concentration values representative of average indoor exposure. Measurements were conducted in residential dwellings during the summer and autumn periods to account for seasonal variability in indoor radon levels associated with differences in ventilation and meteorological conditions. Detectors were placed in living rooms or bedrooms at approximately 1.0–1.5 m above the floor, away from doors, windows, and heating sources, in accordance with standard methodological recommendations. Radon gas concentrations in 55 buildings and dwellings were surveyed over a total exposure time of 3 months in summer (June–August 2025) to autumn (September–December 2025).

Based on measured radon concentrations, individual radon exposure levels were determined for each dwelling. The obtained data were subsequently used to group participants by radon exposure level and to conduct statistical analyses of associations between radon exposure and molecular biomarkers. To better reflect chronic residential exposure, radon concentrations measured during the summer and autumn monitoring periods were combined to calculate an integrated average indoor radon concentration for each dwelling. Exposure classification into high- and low-radon groups was based on the averaged indoor radon concentration using the ICRP reference level of 300 Bq/m^3^. Restriction of participant inclusion to residents living in the investigated areas for at least 5 years further supported the assessment of long-term exposure conditions. Individual radon exposure levels as the annual effective doses derived from the inhalation of indoor radon ($E_{inh}$) were calculated using the following equation suggested by UNSCEAR [1]:

$$E_{inh}=C_{Rn}\times F_{Rn}\times T_{exp}\times {DFC}_{Rn}\times 10^{-6}$$

where $E_{inh}$—the annual effective dose from inhalation of radon (mSv/y); $C_{Rn}$—annual average radon concentration in dwellings (Bq/m^3^); $F_{Rn}$—the indoor equilibrium factor for radon and its respective progenies is 0.40; $T_{exp}$ is the total hours spent indoors annually (h/y), calculated as ($T_{exp}=O_{in}\times 8760,$ where *O_in_* is the indoor occupancy factor (0.8) and 8760 is the number of hours in a year); ${DFC}_{Rn}$ is dose conversion factor for radon = 9 nSv per unit of integrated radon concentration (Bq/(h m^3^)) [16].

### 2.4. Biological Sample Collection

Biological samples were collected from all study participants, including patients with lung cancer and control groups, following standardized and uniform procedures. Sample collection was performed under comparable conditions to minimize preanalytical variability and ensure consistency between study groups.

Venous blood samples (4 mL) were obtained from each participant by trained medical personnel using sterile techniques. In patients with lung cancer, blood sampling was carried out before the initiation of anticancer treatment, when applicable. All participants provided written informed consent for biological sampling and subsequent molecular analyses.

Blood samples were processed immediately after collection according to standard laboratory protocols. The obtained material was aliquoted and stored under appropriate conditions to preserve molecular stability. Samples were stored at −80 °C until further molecular analysis. Sample handling, transportation, and storage were performed in compliance with biosafety and quality control requirements [17].

Each sample was assigned a unique anonymized identification code to ensure confidentiality and enable blinded analysis. The collected blood samples were subsequently used for the assessment of molecular biomarkers associated with radon exposure and lung cancer.

### 2.5. Protein Extraction and Proteomic Analysis

Total protein was extracted from plasma samples according to a modified protocol for plasma protein extraction [18]. Venous blood samples collected in EDTA K2 vacuum tubes were thawed at 4 °C for 12 h and centrifuged at 2000× *g* for 15 min at 4 °C. The plasma-containing supernatant was transferred to clean microcentrifuge tubes. Aliquots of plasma (50 µL) were supplemented with a protease inhibitor cocktail, followed by protein precipitation with cold acetone. Samples were incubated at −20 °C for 1 h and centrifuged at 16,000× *g* for 15 min at 4 °C. The resulting protein pellets were washed with cold acetone, air-dried, and resuspended in 50 mM ammonium bicarbonate buffer (pH 8). In total, 100 plasma protein samples were obtained for further analysis.

Protein concentration was determined using a NanoDrop 1000 spectrophotometer (Thermo Fisher Scientific, Waltham, MA, USA) and a Qubit 4 Fluorometer (Thermo Fisher Scientific, Waltham, MA, USA). For tryptic digestion, protein samples were reduced with dithiothreitol and alkylated with iodoacetamide, followed by overnight digestion with trypsin at 37 °C. After digestion, samples were dried and resuspended in 0.1 mM trifluoroacetic acid. Peptides were purified using C18 reversed-phase columns prior to mass spectrometric analysis. Proteomic analysis was performed using liquid chromatography coupled with tandem mass spectrometry (LC–MS/MS). Peptide separation was carried out on a C18 reversed-phase column using a multistep acetonitrile gradient. Mass spectrometric analysis was conducted on an Impact II ESI-QUAD-TOF mass spectrometer (Bruker Daltonics, Bremen, Germany) equipped with a nano-electrospray ionization source.

Tandem mass spectra were processed by PEAKS 12 software (Bioinformatics Solutions Inc., Waterloo, ON, Canada). The PEAKS 12 database was set up to search the SwissProt human databases (Release 2026_01, 20,432 entries), assuming trypsin as the digestion enzyme. Oxidation of methionine and Acetylation (protein N-term) were set as variable modifications, and carbamidomethylation of cysteine residues as a fixed modification. Mass tolerance thresholds were set at 100 ppm for precursor ions and 0.05 Da for fragment ions. In addition, quantifications were performed with the label-free quantification (LFQ) Q module (Bioinformatics Solutions Inc., Waterloo, ON, Canada). A false discovery rate (FDR) of 1% was set for the peptide-spectrum match. Differentially expressed proteins contained at least 1 unique peptide with *p* < 0.05. The parameters for LFQ were 50 ppm for mass error tolerance and automatic detection of retention time shift tolerance.

### 2.6. Statistical Analysis

Statistical analysis was performed using jamovi software (version 2.6.45). Normality of continuous variables was assessed using the Shapiro–Wilk test. As most continuous variables showed non-normal distribution, data are presented as median and interquartile range (Q1–Q3). Categorical variables are presented as absolute frequencies and percentages. Between-group comparisons of categorical variables were performed using Pearson’s chi-square test; when expected cell counts were <5, Fisher’s exact test was applied to ensure validity of statistical assumptions.

Multivariable logistic regression models were constructed to adjust for potential confounding factors, including age, sex, professional status, exposure to hazardous substances, chronic diseases, and family history of cancer, when appropriate.

All statistical tests were two-sided, and a *p* value < 0.05 was considered statistically significant.

## 3. Results

### 3.1. Characteristics of the Participants

A total of 130 participants were included in the study, comprising 57 individuals in the cancer group and 73 in the control group. The groups were comparable with respect to sex distribution and smoking status (*p* > 0.05). Baseline demographic, clinical, and environmental characteristics of the study participants are summarized in Table 1. Significant differences were observed between the groups in age and duration of residence; due to non-normal distribution of these variables, comparisons were performed using the Mann–Whitney U test (*p* < 0.001 for both variables). The cancer group demonstrated a higher proportion of retired individuals compared with the control group (73.7% vs. 56.2%, *p* = 0.039). Differences in ethnic composition were also statistically significant (*p* = 0.001). No significant differences were found regarding the type of heating used (*p* = 0.157). However, exposure to hazardous chemical substances was more frequently reported in the cancer group (42.1% vs. 21.9%, *p* = 0.013). Chronic diseases were significantly more prevalent among participants in the cancer group compared with controls (54.4% vs. 28.8%, *p* = 0.003). Additionally, a family history of cancer was reported significantly more often in the cancer group (40.4% vs. 12.3%, *p* < 0.001).

### 3.2. Indoor Radon Concentrations and Radiation Exposure

**Figure 2:**
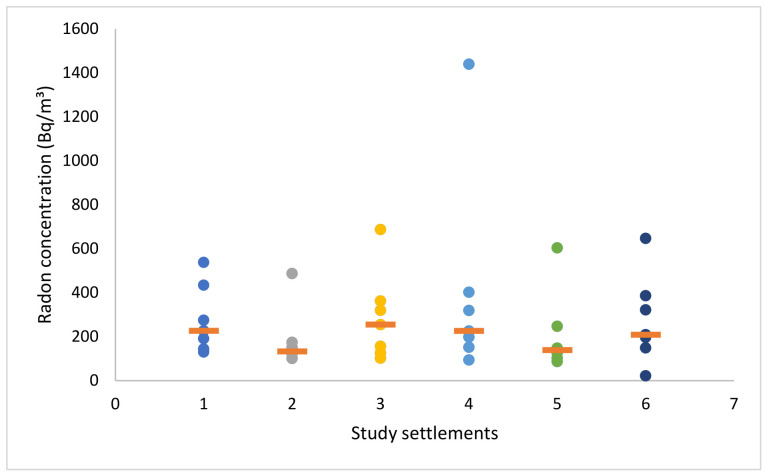
Dot plots of indoor radon concentrations in the summer period across study settlements (1—Aksu; 2—Saumalkol; 3—Atbasar; 4—Burabay-Shuchinsk; 5—Esil; 6—Makinsk). Points represent an individual measurements; horizontal bars indicate median values.

**Figure 3:**
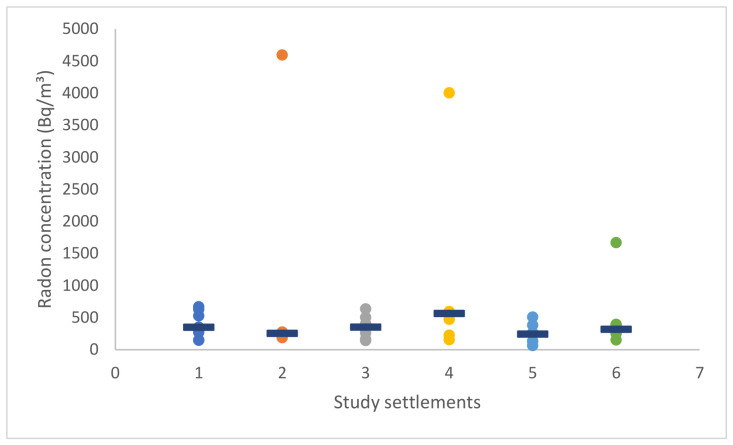
Dot plots of indoor radon concentrations in the autumn period across study settlements (1—Aksu; 2—Saumalkol; 3—Atbasar; 4—Burabay-Shuchinsk; 5—Esil; 6—Makinsk). Points represent individual measurements; horizontal bars indicate median values.

Figure 2 and Figure 3 present the comparison of radon concentration distributions measured in indoor premises in the summer and autumn periods, respectively.

As can be seen from Figure 2, during the summer period, indoor radon concentrations in Makinsk ranged from 150 to 1670 Bq/m^3^, with a mean value of 495 ± 420 Bq/m^3^ and a median of 320 Bq/m^3^. In Burabay, concentrations varied from 80 to 600 Bq/m^3^, with a mean of 380 ± 180 Bq/m^3^ and a median of 470 Bq/m^3^. For Esil, radon concentrations ranged from 65 to 510 Bq/m^3^, with a mean of 245 ± 150 Bq/m^3^ and a median of 245 Bq/m^3^. In Saumalkol, values ranged from 75 to 280 Bq/m^3^, with a mean of 210 ± 70 Bq/m^3^ and a median of 230 Bq/m^3^. In Atbasar, indoor radon concentrations ranged from 145 to 640 Bq/m^3^, with a mean of 350 ± 160 Bq/m^3^ and a median of 352 Bq/m^3^. For Aksu, concentrations ranged from 150 to 670 Bq/m^3^, with a mean of 400 ± 210 Bq/m^3^ and a median of 350 Bq/m^3^.

A comparison of the mean and median values indicates differences in the distribution of radon concentrations among the settlements. In Makinsk, the mean value is considerably higher than the median, suggesting the presence of several high radon measurements that skew the distribution toward larger values. In contrast, in Burabay, the median exceeds the mean, indicating that most measurements are relatively high while a few lower values reduce the average. In Esil and Atbasar, the mean and median values are relatively similar, suggesting a moderately symmetric distribution of radon concentrations. In Saumalkol, the median slightly exceeds the mean, indicating a modest skew toward higher concentrations, whereas in Aksu the mean is higher than the median, reflecting the influence of several elevated radon measurements.

As can be seen from Figure 3, during the autumn period, indoor radon concentrations in Makinsk ranged from 152 to 1668 Bq/m^3^, with a mean value of 496 ± 495 Bq/m^3^ and a median of 319 Bq/m^3^. In Burabay-Shuchinsk, concentrations varied from 156 to 4003 Bq/m^3^, with a mean of 943 ± 1425 Bq/m^3^ and a median of 564 Bq/m^3^. For Esil, radon concentrations ranged from 65 to 509 Bq/m^3^, with a mean of 247 ± 153 Bq/m^3^ and a median of 244 Bq/m^3^. In Saumalkol, values ranged from 187 to 4594 Bq/m^3^, with a mean of 856 ± 1585 Bq/m^3^ and a median of 254 Bq/m^3^. In Atbasar, indoor radon concentrations ranged from 143 to 636 Bq/m^3^, with a mean of 352 ± 184 Bq/m^3^ and a median of 352 Bq/m^3^. For Aksu, concentrations ranged from 147 to 670 Bq/m^3^, with a mean of 392 ± 210 Bq/m^3^ and a median of 350 Bq/m^3^.

During the autumn period, the mean values in Makinsk, Burabay-Shuchinsk, Saumalkol, and Aksu exceed the median values, suggesting the presence of elevated radon measurements that increase the average concentration. The most pronounced differences are observed in Saumalkol and Burabay-Shuchinsk, reflecting the influence of extremely high radon levels recorded in several dwellings. In contrast, in Atbasar, the mean and median values are identical, indicating a relatively symmetric distribution of radon concentrations without strong outliers. In Esil, the mean and median values are also very similar, suggesting moderate variability without extreme deviations.

The estimated effective dose from inhalation of indoor radon varied among the studied settlements and between the summer and autumn monitoring periods. During the summer period, the estimated effective doses were 7 mSv/y in Aksu, 5 mSv/y in Saumalkol, 7 mSv/y in Atbasar, 10 mSv/y in Burabay, 5 mSv/y in Esil, and 7 mSv/y in Makinsk. During the autumn period, the corresponding values increased to 10 mSv/y in Aksu, 22 mSv/y in Saumalkol, 9 mSv/y in Atbasar, 24 mSv/y in Burabay, 6.2 mSv/y in Esil, and 12 mSv/y in Makinsk. These findings indicate pronounced seasonal variability in radon-related radiation exposure, with substantially higher effective doses observed in the autumn period in several settlements, particularly in Saumalkol and Burabay.

### 3.3. Proteomic Analysis of Plasma Samples

#### 3.3.1. Plasma Proteome Differences Between Lung Cancer Patients and Control Group

A total of 906 proteins were identified across all analyzed plasma samples using LC–MS/MS. Of these, 243 proteins were detected in both control individuals and lung cancer patients, whereas 349 proteins were identified exclusively in plasma from lung cancer patients, and 314 proteins were detected only in the control group. Further analysis focused on a subset of core proteins present in both groups. A false discovery rate (FDR) of 1% was applied at the peptide-spectrum match (PSM) level.

**Figure 4:**
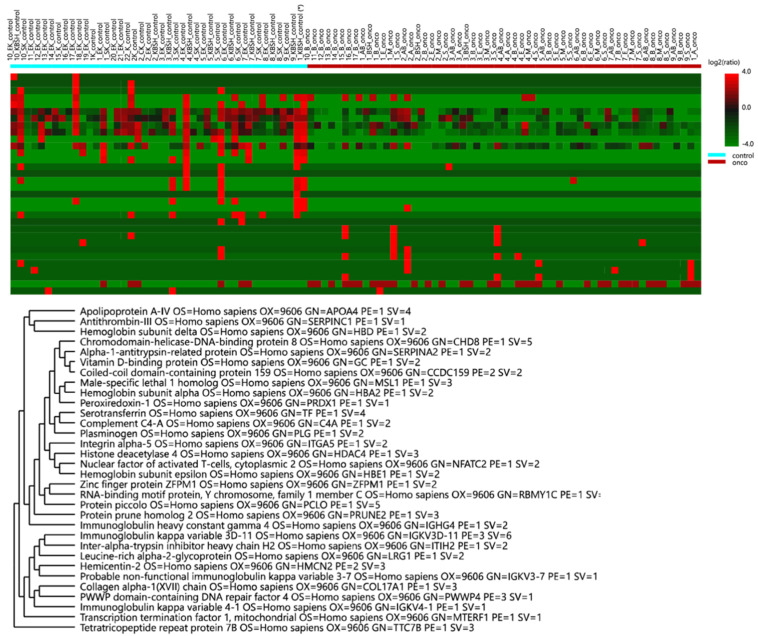
Heatmap illustrating the differential expression of plasma proteins in control individuals and lung cancer patients. Protein profile heatmap generated from PEAKS Studio quantitation module, showing significantly differentially expressed proteins following quantitative LC-MS/MS analysis utilizing the Top 3 peptides from each protein. Cell color represents the log2(ratio) to the average area across different samples. (*) is the base sample for retention time alignment and feature matching.

**Figure 5:**
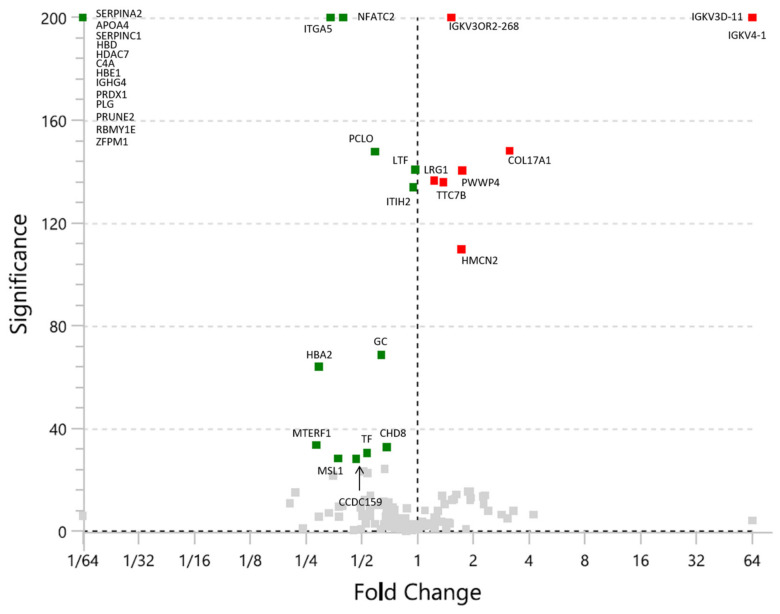
Volcano plot of differentially expressed plasma proteins detected in both control and lung cancer groups. The *x*-axis represents fold change, and the *y*-axis indicates statistical significance (−log10 *p*-value). Proteins with increased expression in the lung cancer group are shown in red, while proteins with decreased expression are shown in green.

Label-free quantitation (LFQ) analysis identified 42 proteins in both groups that exhibited differential abundance (Figure 4 and Figure 5). Differentially abundant proteins were defined as those identified with at least one unique peptide and meeting a statistical significance threshold of *p* < 0.05. After exclusion of highly abundant plasma proteins, including albumin, hemoglobin, collagen, and immunoglobulins, 13 proteins common to both sample groups were retained. These proteins showed statistically significant differences between lung cancer patients and controls (Table 2).

Principal component analysis (PCA) demonstrating proteomic profile separation between lung cancer patients and controls is presented in Supplementary Figure S1.

#### 3.3.2. Plasma Proteome Differences Between Lung Cancer Patients and Radon-Exposed Lung Cancer Patients

To assess the potential impact of radon exposure on the plasma proteome of lung cancer patients, a comparative LFQ analysis was performed using 20 plasma samples from lung cancer patients residing in dwellings with elevated indoor radon concentrations (>300 Bq/m^3^; 811.7 ± 977.0 Bq/m^3^) and 37 plasma samples from lung cancer patients residing in dwellings with lower indoor radon concentrations (<300 Bq/m^3^; 188 ± 72 Bq/m^3^). Radon exposure classification was based on CR-39 measurements of indoor radon concentrations in residential buildings, combining data obtained during summer and autumn periods. Data processing and statistical analysis were performed in PEAKS Studio 12.

Principal component analysis (PCA) demonstrating proteomic profile differences between radon-exposed and low-exposure lung cancer patients is presented in Supplementary Figure S2.

**Figure 6:**
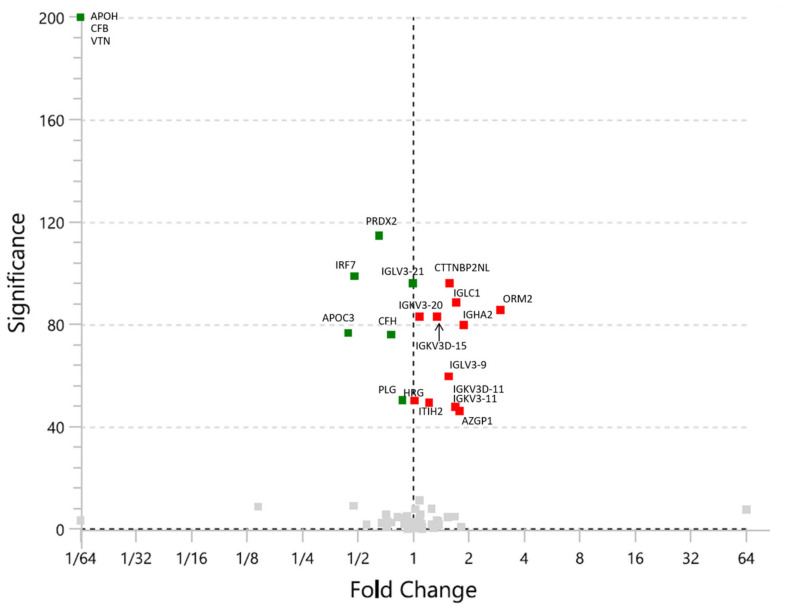
Volcano plot of radon-associated differentially expressed plasma proteins. The *x*-axis represents fold change, and the *y*-axis indicates statistical significance (−log10 *p*-value). Each point corresponds to an individual protein. Proteins with increased expression associated with radon exposure are shown in red, whereas proteins with decreased expression are shown in green. The vertical dashed line denotes a fold change of 1, indicating no difference in protein expression relative to the reference level.

**Figure 7:**
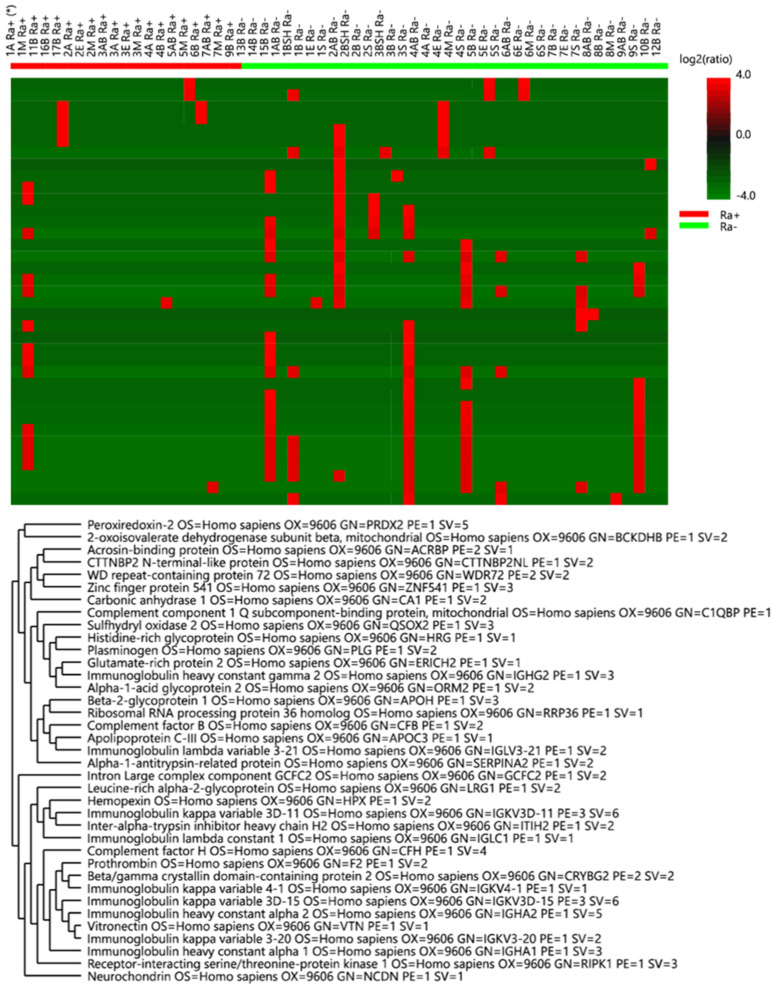
Heatmap of differentially expressed plasma proteins distinguishing radon-exposed and non-exposed lung cancer patients. Protein profile heatmap generated from PEAKS Studio quantitation module, showing significantly differentially expressed proteins following quantitative LC-MS/MS analysis utilizing the Top 3 peptides from each protein. Cell color represents the log2(ratio) to the average area across different samples. (*) is the base sample for retention time alignment and feature matching.

Following initial analysis, 101 proteins were identified in both the control group and lung cancer patients. A false discovery rate (FDR) of 1% was applied at the peptide-spectrum match (PSM) level. A final set of 21 proteins that demonstrated differential expression in both radon-exposed and non-exposed groups (Figure 6 and Figure 7). After removal of highly abundant plasma proteins, including hemoglobin, albumin, and immunoglobulins, 10 proteins common to both groups were retained (Table 3).

Among these, three proteins showed more than a 1.5-fold increase in plasma from radon-exposed lung cancer patients, including ORM2, AZGP1, and CTTNBP2N. In contrast, three proteins (PRDX2, IRF7, and APOC3) demonstrated more than a 1.5-fold lower expression in lung cancer patients with low radon exposure. The overall distribution of radon-associated proteomic differences is illustrated in the volcano plot, with quantitative values summarized in Table 3.

## 4. Discussion

Indoor radon concentrations measured in the present study were compared with internationally recommended reference levels to assess potential health risks. According to the World Health Organization (WHO), the recommended reference level for indoor radon concentration is 100 Bq/m^3^, while the International Commission on Radiological Protection (ICRP) recommends a reference level of 300 Bq/m^3^ for existing dwellings [3,18]. The results of the present study indicate that a considerable proportion of measured indoor radon concentrations exceeded recommended reference levels. In particular, radon concentrations above 300 Bq/m^3^ were observed in more than 50% of dwellings in Makinsk, Burabay-Shuchinsk, Atbasar, and Aksu. In Esil, approximately 20% of measurements exceeded 300 Bq/m^3^. In contrast, in Saumalkol, radon concentrations did not exceed 300 Bq/m^3^ during the summer monitoring period; however, elevated values were observed during the autumn period, with the maximum concentration reaching 3040 Bq/m^3^, substantially exceeding both WHO and ICRP reference levels. Even in settlements with lower average radon concentrations, several measurements exceeded the WHO reference level of 100 Bq/m^3^, indicating potential radon exposure risks for residents. Even in settlements with lower average concentrations, such as Esil, individual measurements exceeded 100 Bq/m^3^, indicating potential radon exposure risks for residents. These findings suggest that indoor radon exposure in several studied settlements may represent a potential environmental health concern and highlight the importance of continuous radon monitoring and implementation of mitigation measures in residential buildings.

In addition to environmental assessment, the present study investigated molecular biomarkers associated with lung cancer using plasma proteomic analysis. Differences in circulating protein expression profiles were observed between lung cancer patients and control individuals, as well as between radon-exposed and lower-exposure groups. Several identified proteins are involved in biological pathways related to inflammation, lipid metabolism, complement activation, and coagulation processes, which are known to play important roles in tumor initiation and progression [19]. As a result of differential expression analysis, ORM2, AZGP1, and CTTNBP2N proteins exhibited the most pronounced upregulation in radon-exposed lung cancer patients, whereas PRDX2, IRF7, and APOC3 proteins were downregulated in patients with low radon exposure (<300 Bq/m^3^).

Differential expression of plasma proteins identified in this study reflects biological processes associated with inflammation, immune regulation, lipid metabolism, oxidative stress, and extracellular matrix remodeling, all of which are known to contribute to lung carcinogenesis.

Proteins involved in inflammatory and acute-phase responses, including ORM2, HRG, and LRG1, indicate activation of systemic inflammatory pathways frequently observed in cancer patients. LRG1 has been associated with angiogenesis and modulation of TGF-β signaling, contributing to tumor progression and metastasis. Chronic inflammation is recognized as a key contributor to tumor initiation and progression through induction of genomic instability, stimulation of angiogenesis, and modulation of immune responses [20,21]. Elevated levels of acute-phase proteins have been reported in lung cancer and may reflect tumor-associated immune activation. Alterations in proteins associated with lipid metabolism, including APOC3 and AZGP1, may reflect metabolic reprogramming characteristic of malignant cells. Tumor cells require increased lipid synthesis to support membrane formation, energy production, and proliferation [22]. Dysregulation of lipid transport proteins has been previously observed in lung cancer patients, supporting their potential role as circulating biomarkers.

Several identified proteins are related to extracellular matrix remodeling, complement activation, and coagulation pathways, including ITIH2, PLG, and CFH. Dysregulation of these pathways has been linked to tumor invasion, angiogenesis, and metastatic dissemination. Changes in adhesion-related proteins such as ITGA5 further support the involvement of cell–matrix interaction pathways in lung cancer progression [23,24,25].

Proteins associated with oxidative stress and immune signaling, including PRDX2 and IRF7, suggest activation of cellular stress response pathways. Oxidative stress contributes to DNA damage and may promote malignant transformation through the accumulation of genetic alterations [26,27].

Although both tobacco smoking and radon exposure contribute to oxidative stress and lung carcinogenesis, the underlying molecular mechanisms differ substantially. Radon progeny emit high-linear energy transfer (high-LET) alpha particles that induce localized clustered DNA damage, persistent reactive oxygen species generation, and chronic inflammatory signaling. In contrast, tobacco-related oxidative stress is primarily associated with exposure to chemical carcinogens that activate xenobiotic metabolism and detoxification pathways. Altered expression of proteins such as PRDX2 and IRF7 observed in the present study may therefore reflect molecular responses associated more specifically with radiation-induced oxidative injury and immune dysregulation.

Some of the observed proteomic alterations may also reflect biological effects associated with radon exposure. Alpha-particle radiation emitted by radon decay products induces oxidative stress, DNA damage, and inflammatory signaling, potentially influencing circulating protein expression patterns [28]. These findings support the hypothesis that plasma protein biomarkers may provide insight into molecular mechanisms linking environmental exposure and lung carcinogenesis.

Data regarding radon-associated proteomic alterations in populations residing near uranium legacy sites remain limited, particularly in Central Asia. The present study integrates environmental radon exposure assessment with plasma proteomic profiling in lung cancer patients from Kazakhstan, thereby providing novel regional evidence regarding potential molecular mechanisms of radon-associated carcinogenesis. These findings may contribute to future development of exposure-related biomarker panels and improve the understanding of environmental risk factors involved in lung cancer progression.

Overall, the identified proteins represent biologically plausible candidates associated with inflammation, metabolic adaptation, and tumor microenvironment remodeling, supporting their potential relevance as circulating biomarkers of lung cancer.

One limitation of the present study is the relatively small sample size for proteomic analysis, which may limit the generalizability of the identified candidate biomarkers. In addition, proteomic changes may be influenced by multiple factors, including smoking status, age, and comorbidities. Further studies involving larger patient cohorts and targeted validation of identified proteins are necessary to confirm their clinical relevance and potential application in early detection, risk assessment, and monitoring of lung cancer associated with environmental radon exposure [29,30,31].

The present study should be considered an exploratory investigation providing preliminary insight into proteomic alterations associated with radon exposure and lung cancer in populations residing near uranium legacy sites in Kazakhstan. Future studies involving larger independent cohorts, longitudinal follow-up, targeted validation assays, and advanced statistical modeling will be performed to further evaluate the diagnostic and biological relevance of the identified candidate biomarkers.

## 5. Conclusions

This study investigated the relationship between indoor radon exposure and lung cancer risk in the northern regions of Kazakhstan by combining environmental measurements with molecular biomarker analysis. The results demonstrated elevated indoor radon concentrations in several settlements, indicating potential environmental risk factors for lung cancer development. In addition to environmental monitoring, proteomic analysis revealed differences in circulating plasma proteins between lung cancer patients and control individuals, as well as between radon-exposed and non-exposed patients. Several identified proteins were associated with inflammatory response, lipid metabolism, and coagulation pathways, which are known to play important roles in tumor development and progression. These findings suggest that radon exposure may contribute not only to increased lung cancer risk but also to molecular alterations detectable in circulating proteins. Although further validation in larger cohorts is required, the integration of environmental assessment and proteomic profiling may provide new opportunities for understanding radon-related carcinogenesis and improving early detection strategies.

## Figures and Tables

**Table 1:** Presents the baseline demographic, clinical, and environmental characteristics of the study participants.

Variable	Cancer Group (*n* = 57)	Control Group (*n* = 73)	*p* Value
**Sex (%)**			0.241
Male	34 (59.6)	36 (49.3)	
Female	23 (40.4)	37 (50.7)	
**Age category (median category)**	61–70 years	51–60 years	<0.001 *
**Duration of residence category (median residence category)**	lifelong residence	11–20 years	<0.001 *
**Ethnicity, *n* (%)**			0.001
Kazakh	16 (28.1)	42 (57.5)	
Russian	27 (47.4)	25 (34.2)	
Other (Ukrainian, Germany)	14 (24.6)	6 (8.2)	
**Occupation, *n* (%)**			0.039
Retired	42 (73.7)	41 (56.2)	
Employed	15 (26.3)	32 (43.8)	
**Ever smoked, *n* (%)**	22 (38.6)	26 (35.6)	0.727
**Central heating, *n* (%)**	24 (42.1)	41 (56.2)	0.157
**Exposure to hazardous substances, *n* (%)**	24 (42.1)	16 (21.9)	0.013
**Chronic diseases, *n* (%)**	31 (54.4)	21 (28.8)	0.003
**Family history of cancer, *n* (%)**	23 (40.4)	9 (12.3)	<0.001

**Note:** Age categories were defined as: 0 = ≤40 years, 1 = 41–50 years, 2 = 51–60 years, 3 = 61–70 years, 4 = 71–80 years. Duration of residence categories was defined as: 0 = ≤10 years, 1 = 11–20 years, 2 = 21–40 years, 3 = lifelong residence. * Statistically significant according to the Mann–Whitney U test.

**Table 2:** Differential expression of plasma proteins in lung cancer patients versus controls.

Protein	Gene	Significance	Expression Ratio (Control: Cancer); Fold Change	Molecular Weight (Da)
PWWP domain-containing DNA repair factor 4	*PWWP4*	140.30	1.00:1.75; 1.75	217,773
Hemicentin-2	*HMCN2*	109.73	1.00:1.73; 1.73	541,978
Leucine-rich alpha-2-glycoprotein	*LRG1*	136.60	1.00:1.23; 1.23	38,178
Inter-alpha-trypsin inhibitor heavy chain H2	*ITIH2*	133.83	1.00:0.95; −1.05	106,463
Chromodomain-helicase-DNA-binding protein 8	*CHD8*	32.63	1.00:0.68; −1.47	290,519
Vitamin D-binding protein	*GC*	68.55	1.00:0.64; −1.56	52,918
Protein piccolo	*PCLO*	147.78	1.00:0.59; −1.69	560,699
Serotransferrin	*TF*	30.48	1.00:0.53; −1.87	77,050
Coiled-coil domain-containing protein 159	*CCDC159*	28.20	1.00:0.47; −2.14	33,695
Nuclear factor of activated T-cells, cytoplasmic 2	*NFATC2*	200.00	1.00:0.40; −2.52	100,146
Male-specific lethal 1 homolog	*MSL1*	28.25	1.00:0.37; −2.68	67,128
Integrin alpha-5	*ITGA5*	200.00	1.00:0.34; −2.95	114,536
Transcription termination factor 1, mitochondrial	*MTERF1*	33.50	1.00:0.29; −3.50	45,778

**Table 3:** Differentially expressed plasma proteins identified by proteomic analysis in lung cancer patients with radon compared with lung cancer patients without radon exposure.

Protein	Gene	Significance	Expression Ratio (Lung Cancer Ra Positive: Lung Cancer Rad Negative); Fold Change	Molecular Weight (Da)
Alpha-1-acid glycoprotein 2	*ORM2*	85.57	2.96:1.00; 2.96	23,603
Zinc-alpha-2-glycoprotein	*AZGP1*	45.98	1.78:1.00; 1.78	34,259
CTTNBP2 N-terminal-like protein	*CTTNBP2NL*	96.05	1.58:1.00; 1.58	70,158
Inter-alpha-trypsin inhibitor heavy chain H2	*ITIH2*	49.23	1.22:1.00; 1.22	106,463
Histidine-rich glycoprotein	*HRG*	50.14	1.01:1.00; 1.01	59,578
Plasminogen	*PLG*	50.27	0.87:1.00; −1.15	90,569
Complement factor H	*CFH*	76.01	0.76:1.00; −1.32	139,096
Peroxiredoxin-2	*PRDX2*	114.54	0.65:1.00; −1.54	21,892
Interferon regulatory factor 7	*IRF7*	98.73	0.48:1.00; −2.08	54,278
Apolipoprotein C-III	*APOC3*	76.55	0.44:1.00; −2.27	10,852

## Data Availability

The mass spectrometry proteomics data have been deposited to the ProteomeXchange Consortium via the MassIVE repository partner repository with the dataset identifier PXD076702.

## Supplementary Materials

The following supporting information can be downloaded at https://www.mdpi.com/article/10.3390/biomedicines14061204/s1, Figure S1. Principal component analysis (PCA) plot demonstrating group-level proteomic profile differences between lung cancer patients and the control group; Figure S2. Principal component analysis (PCA) plot demonstrating group-level proteomic profile differences between radon-exposed (RA+) and low-exposure (RA−) lung cancer patients.

## Abbreviations

The following abbreviations are used in this manuscript:

Rn	Radon
WHO	World Health Organization
ICRP	International Commission on Radiological Protection
CR-39	Solid-state nuclear track detector
LFQ	Label-Free Quantification
LC–MS/MS	Liquid Chromatography–Tandem Mass Spectrometry
SNP	Single Nucleotide Polymorphism
DNA	Deoxyribonucleic acid
LET	Linear Energy Transfer
ORM1	Orosomucoid 1
SERPINA1	Serpin Family A Member 1
APOA1	Apolipoprotein A1
APOA2	Apolipoprotein A2
APOC3	Apolipoprotein C3
FGA	Fibrinogen Alpha Chain
FGB	Fibrinogen Beta Chain
FGG	Fibrinogen Gamma Chain
*TF*	Transferrin
OR	Odds Ratio
CI	Confidence Interval
