# Supplementary material for: Identification of Novel Protein Biomarkers for Early Detection of Radon-Induced Lung Cancer: A Comparative Study in Kazakhstan
Source: Biomedicines. 2026 May 27;14(6):1204. doi: 10.3390/biomedicines14061204 (PMC13297576; doi:10.3390/biomedicines14061204)
Supplement: Supplementary file 1 [file biomedicines-14-01204-s001.zip › Supplementary materials_compressed.pdf]

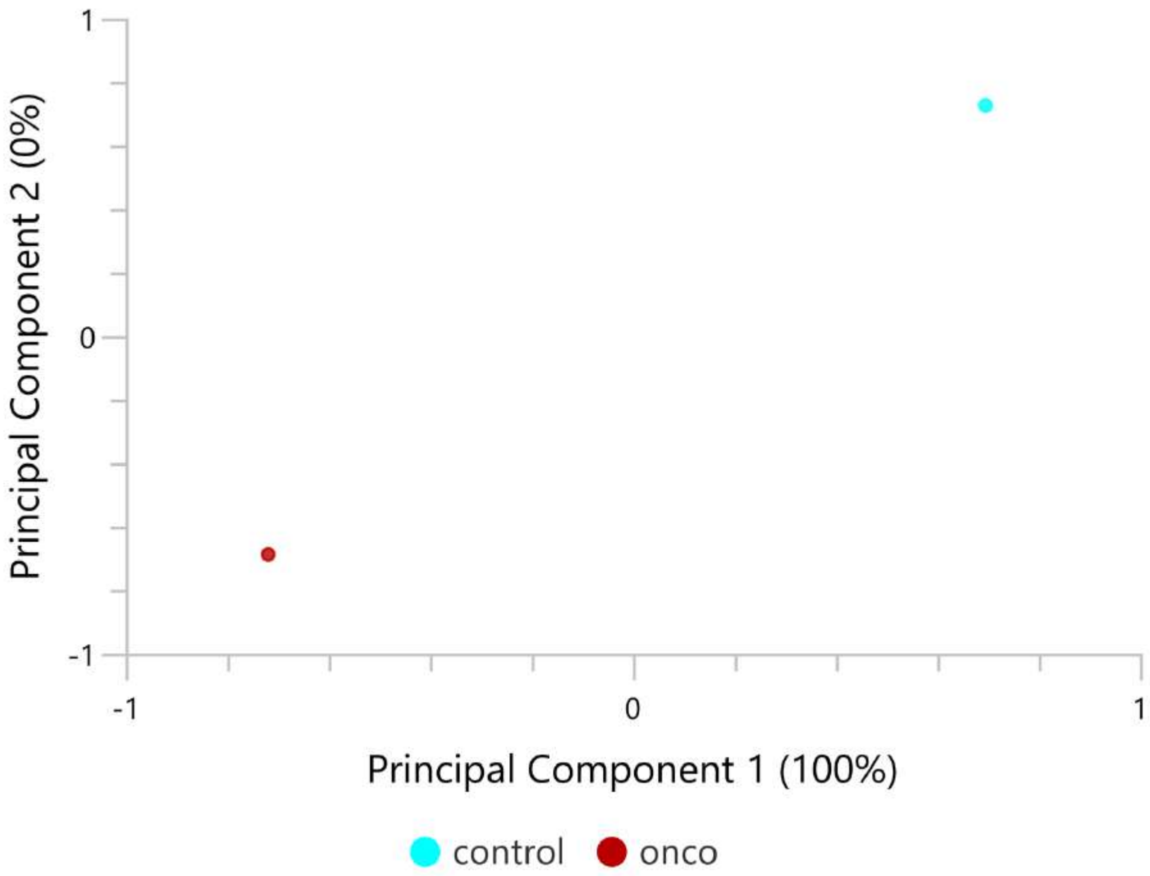

Principal component analysis (PCA) plot demonstrating group level proteomic profile differences between lung cancer patients and control group

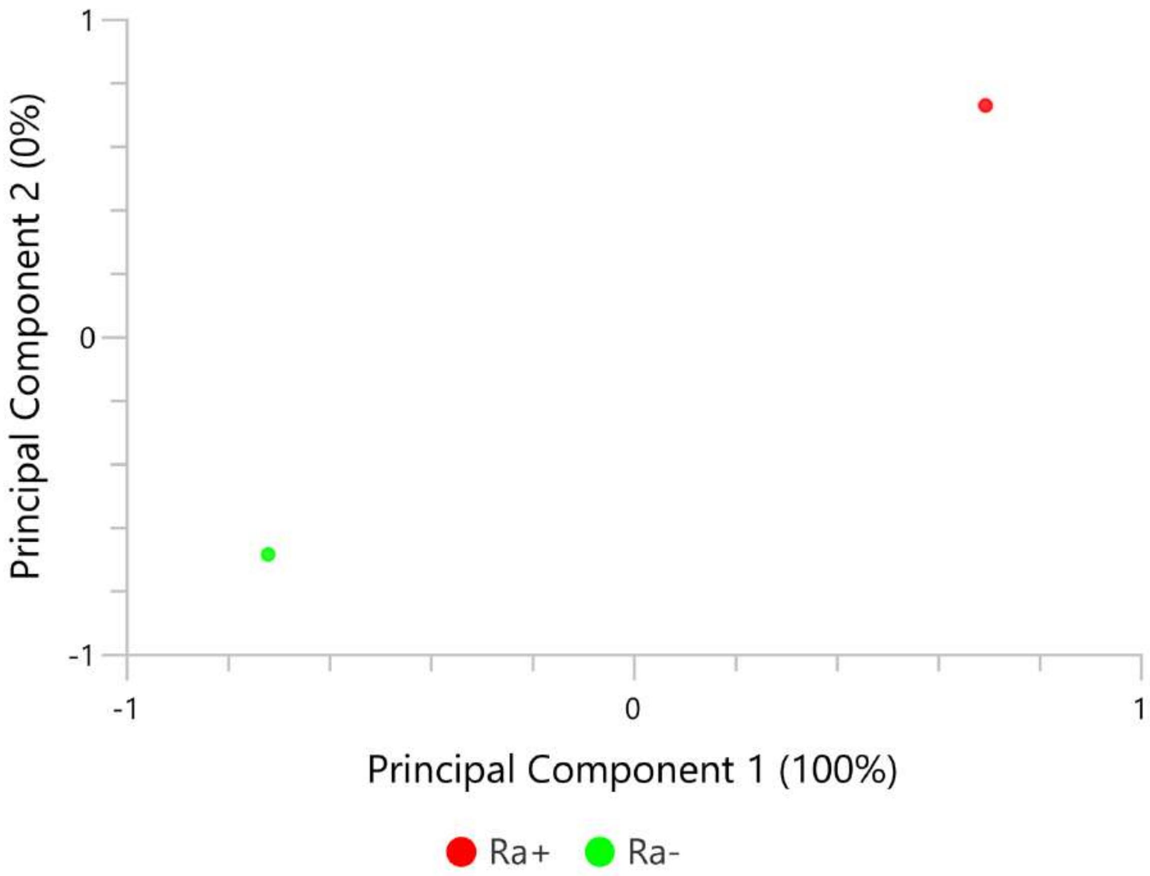

Principal component analysis (PCA) plot demonstrating group level proteomic profile differences between radon exposed (RA+) and low exposure (Ra-) lung cancer patients
