# Supplementary figures and images for: Identification of Novel Protein Biomarkers for Early Detection of Radon-Induced Lung Cancer: A Comparative Study in Kazakhstan
Source: Biomedicines. 2026 May 27;14(6):1204. doi: 10.3390/biomedicines14061204 (PMC13297576; doi:10.3390/biomedicines14061204)

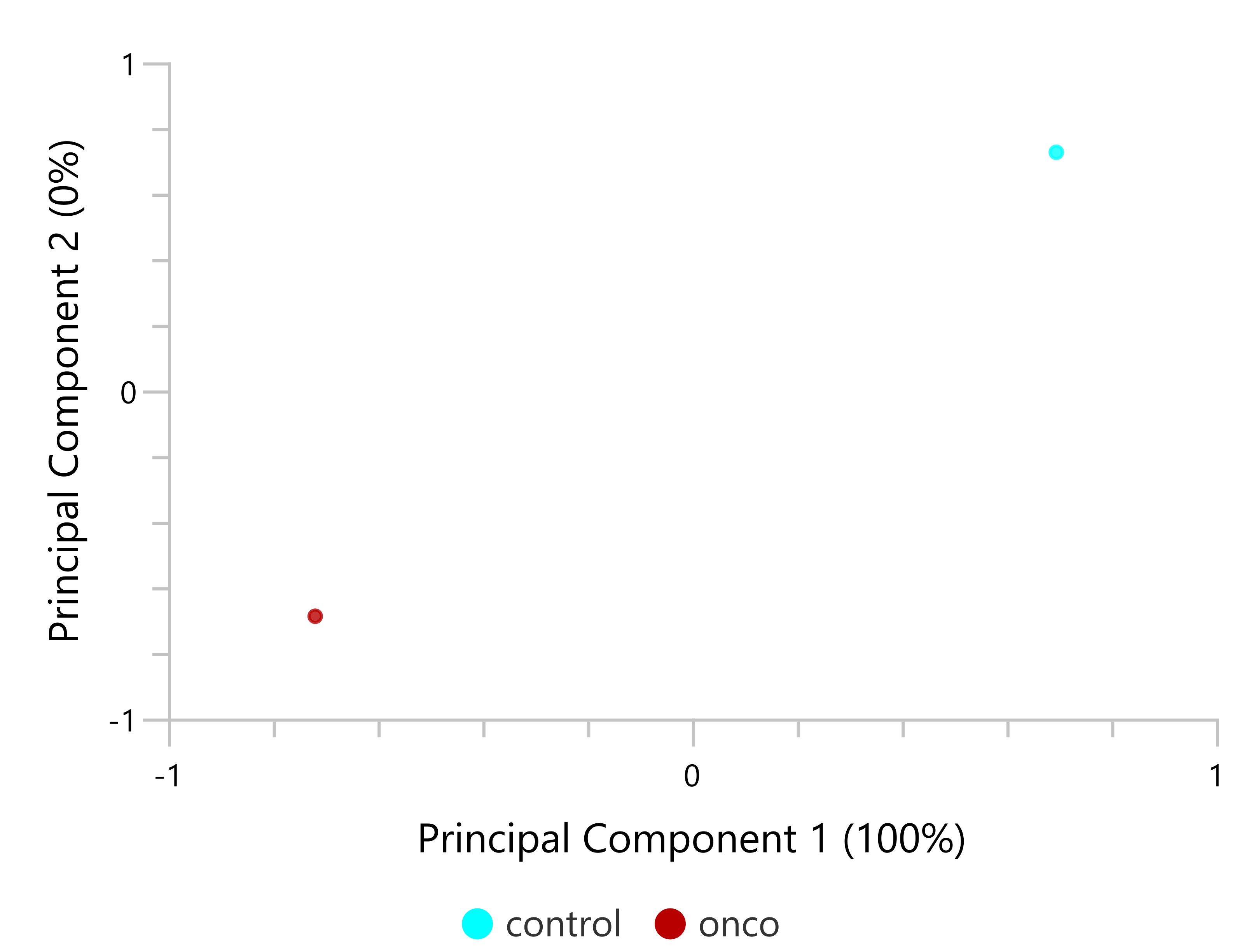

Supplement: Supplementary file 1 [file biomedicines-14-01204-s001.zip › 100_PCA-Plot-By-Group-.tif]

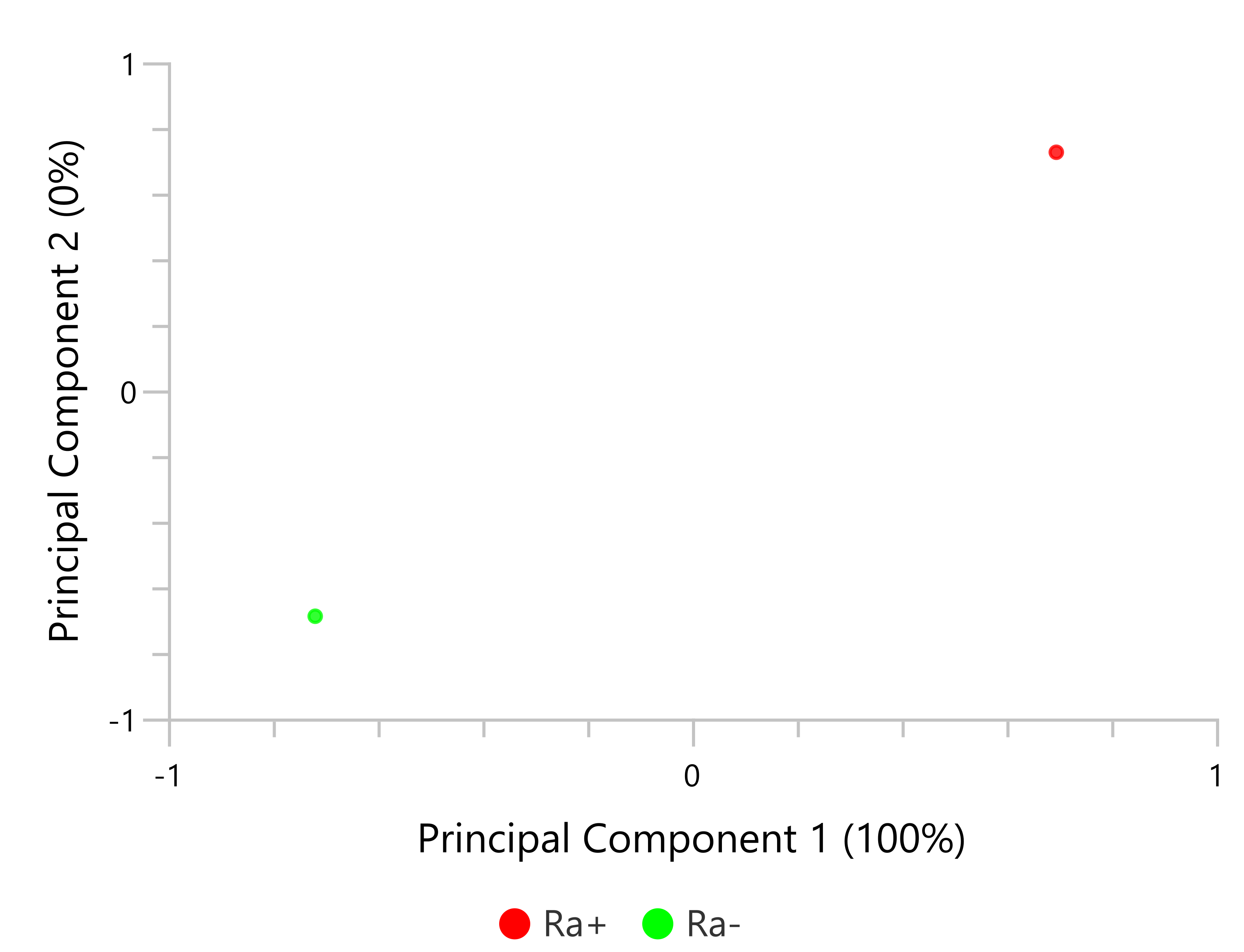

Supplement: Supplementary file 1 [file biomedicines-14-01204-s001.zip › 57_PCA-Plot-By-Group-.tif]
